# Chitin-Functionalized Carbon Nanofiber-Based Electrochemical Sensor for Rapid and Sensitive Detection of 4‑Methylaminophenol in Aquatic Ecosystems

**DOI:** 10.1021/acsomega.5c08044

**Published:** 2025-10-14

**Authors:** Gomathisankar Palavesanarayanan, Balamurugan Arumugam, Rajendran Surya, Krishnan Venkatesh, Subramanian Sakthinathan, Te-Wei Chiu, Sayee Kannan Ramaraj

**Affiliations:** † PG & Research Department of Chemistry, 29967 Thiagarajar College, Madurai, Tamil Nadu 625009, India; ‡ Department of Materials and Mineral Resources Engineering, 34877 National Taipei University of Technology, No.1, Section 3, Chung-Hsiao East Road, Taipei 106, Taiwan; § Institute of Materials Science and Engineering, 34877 National Taipei University of Technology, No. 1, Section 3, Chung-Hsiao East Road, Taipei 106, Taiwan; ∥ Department of Chemistry, National Sun Yat-sen University, No. 70, Lien-hai Road, Kaohsiung 804201, Taiwan; ⊥ Department of Chemistry, Kyungpook National University, Daegu 41566, South Korea

## Abstract

4-Methylaminophenol (4-MAP), commonly used in hair dye and photography industries, has a major undesirable ecological effect owing to its toxicity, persistence, and bioaccumulation in aquatic environments. Even at low concentrations, 4-MAP poses a threat to aquatic life and human health, and therefore accurate detection is essential as an environmental indicator. Nevertheless, the current approaches are either insensitive enough or too complicated to be applied regularly. In this work, we fabricated a chitin-functionalized carbon nanofiber composite (chitin/f-CNF) using ultrasonication technique and characterized using XRD, FT-IR, Raman, FE-SEM, EDX, elemental mapping, and HR-TEM analyses. The composite was applied to modify a glassy carbon electrode (GCE) and employed in the electrochemical detection of 4-MAP using cyclic voltammetry and differential pulse voltammetry. The chitin/f-CNF modified GCE demonstrates improved electron transfer and electrocatalytic activity compared to other modified and unmodified electrodes. The developed sensor achieved high sensitivity (1.867 μA μM^–1^ cm^–2^), a wide linear range (0.01–747.19 μM), a low detection limit (4.2 nM), and a low quantification limit (14.15 nM). Computational density functional theory simulations further elucidated the electronic energy landscape and charge-transport pathways of 4-MAP during the electrochemical process. The sensor also exhibited excellent selectivity in the presence of potential interfering substances, along with remarkable reproducibility, stability, and repeatability. Real water samples display high recoveries with an accuracy of 97.93% (tap), 99.03% (pond), and 98.93% (river). Overall, the proposed chitin/f-CNF modified GCE demonstrates a simple, inexpensive, and reliable sensitive analysis of 4-MAP which will improve environmental pollution and water quality management.

## Graphical abstract

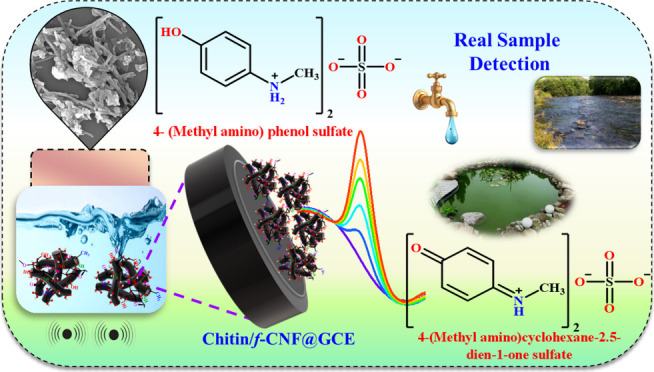

## 1 Introduction

Environmental analysis of organic pollutants from manufacturing industries or internal disposal methods requires more attention due to their effects on ecosystems as well as human health status. Hence, the identification and quantitative determination of organic pollutants remain essential for biomedical diagnostics, environmental monitoring, and industrial safety.^1^ Metol, also known as *N*-methyl-*p*-aminophenol sulfate, exhibits an organic chemical structure with amine and hydroxyl groups up to now it exists commercially as sulfate salt [HO­C_6_­H_4_­NH_2_­(CH_3_)]­HSO_4_ due to its light-induced and air-sensitive nature.^2–4^ Applications of 4-methylaminophenol (4-MAP) include the development of monochrome photographs, corrosion inhibition, polymers, antioxidant properties, and antibacterial agents. 4-MAP plays two roles: it is used in the synthesis of the drug diloxanide and significantly contributes to the coloring of hair and fur.^5,6^ However, 4-MAP from industrial wastes leads to major pollution of environmental ecosystems.^7,8^ It enters water systems primarily through wastewater discharge from both nonrecyclable and industrial operations after the picture processing and hair coloring measures.^9^ High concentrations of this substance create various health-related issues, including hypertension, angina, diarrhea, skin rashes, lung shortness, and neurological disorders.^10^ Drinking water regulation by Chinese authorities includes maximum allowable parameters of 0.002 mg/L for phenolic-based chemicals and 0.02 mg/L for all other aromatic compounds. 4-MAP stays in the environment largely because its limited water solubility blocks biodegradation mechanisms.^11^ There is an urgent need to detect 4-MAP and assess the dangerous impacts on both the ecosystem and human health.^12^ Multiple analytical methods exist to detect amine derivatives through combinations of mass chromatography, HPLC alongside photolysis, positron discharge tomography, and flow injection methods.^13^ Laboratory protocols require specialized equipment and are time consuming and expensive, as noted in previous reports.^14,15^ Among them, electrochemical techniques are inexpensive, simple to operate, and sensitive and can precisely detect analytes at trace levels.^16,17^ The electrochemical sensors are heavily reliant on the qualities of nanomaterials and their combinations utilized to modify the surface of electrodes, which greatly enhance their performances, such as electrochemical redox reaction, electron-transport kinetics, active surface area, limit of detection, sensitivity, and selectivity.^18,19^

Carbon-based nanocomposites have drawn great consideration because of their intrinsic properties such as high conductivity, enhanced surface area, robust π-π interaction with synergistic recombination, flexibility in nanocomposite preparation, and mechanical and thermal stabilities. These properties make them extremely applicable in a number of applications, specifically in energy storage, sensors, and catalysis. They are not only important due to their physical properties but also may find new innovative uses, including in nanosensors that can transform technologies in the field of detection. Chitin is a biopolymer that originates from the hard shells of crustaceans, including crabs and shrimps. It is rich in β(1→4)-linked glycan bonds, which is the second most abundant biopolymer after cellulose. Chitin contains 2-acetamido-2-deoxy-β-d-glucose (*N*-acetyl glucosamine) with a molecular weight higher than one million.^20,21^ Crab shell-derived chitin powder is an affordable and environmentally friendly carbon material that can be used as a promising candidate in electrode materials and electrochemical applications. Chitin possesses various necessary features including high renewability, biocompatibility, mechanical stability, and biodegradability, as well as the functional groups that can easily make contact with different molecules.^22^ These properties allow chitin to produce new structures with capacity (flexibility) to form fibers, hydrogels, beads, sponges, and membranes along with scarce physicochemical characteristics.^23^ Chitin-based materials have been used in a wide range applications including multiple protein sorption. It serves as a shale inhibitor in aquatic drilling liquids while maintaining prospects for stress reduction of phenolic compounds, food packaging, energy-related functions such as nanogenerators and batteries,^24–27^ photodegradation,^28^ water treatment,^29^ and medical applications including tissue engineering, emulsion development, wound care solutions, and drug-delivery systems.^30,31^ Also, the use of chitin powder or other carbon nanomaterials as surface modifiers significantly enhances the conductivity and active surface area of the interface layer. It shortens the ion diffusion path and amplifies the chemical stability, which improves the electron-transport efficiency between the modified electrode and the targeted analyte in electrolyte solution.

In electrochemical applications, carbon nanofibers (CNFs) are another notable promising carbon materials for electrode modifiers owing to their superior physical-chemical properties including nontoxicity, inexpensive, defect-rich sites, superior electrical conductance, chemical stability, stacking arrangement of layers, and enlarged surface area. Due to these properties, CNFs have been used in major current research fields, including electrochemical sensors, photocatalysis, supercapacitors, and batteries. Functionalization of CNFs further increases their physical and chemical properties with the inclusion of various edge-plane defect sites that enhance electron-donor ability. Here, we acid hydrolyzed and partially oxidized the functional groups of the CNFs to yield f-CNFs with (−COOH, −OH, and −COO) groups, which significantly enhance their reactivity, surface chemistry, and solubility that are favorable for electrode modifications.^32^ Combining these two carbon materials, chitin and f-CNFs, formed a chitin/f-CNF nanocomposite that displays outstanding properties such as effective ion-transport channel, rich electron-transfer rates, large surface area, electrical conductivity, and enhanced charge diffusion pathways. Synergistic interaction between chitin and f-CNFs makes it an excellent electrocatalyst, particularly for the determination of 4-MAP, which highlights the potential of carbon-based nanocomposites in promoting the electrochemical uses especially in sensor technology. Previously, various electrochemical sensors based on carbon interacted nanocomposites have been developed for the detection of 4-MAP, demonstrating excellent selectivity, sensitivity, and broad linear ranges. For example, functionalized halloysite nanotubes (f-HNTs) exhibit a linear range of 0.01–480 μM and a detection limit of 2.1 nM,^5^ while another sensor offers an even wider linear range of 0.005–689 μM and an ultralow detection limit of 0.72 nM.^12^ Reduced graphene oxide (rGO)-based sensors provide a dynamic linear response range of 0.01–137.65 μM with a detection limit of 0.050 μM.^14^ Despite these advancements, most sensors have linear ranges limited to approximately 1–100 μM, and their detection limits, typically in the μM range, remain insufficient for accurate detection of 4-MAP in trace levels.

To address these challenges, this study aims to prepare an innovative chitin/f-CNF nanocomposite by a simple, sustainable sonochemical approach. The physicochemical properties were subsequently characterized using various techniques such as X-ray diffraction (XRD), FT-IR, Raman, FE-SEM, HR-TEM, energy-dispersive spectroscopy (EDX), and elemental mapping. Upon confirming the nanocomposite properties, it was drop-cast onto the surface of a glassy carbon electrode (GCE) to develop a sensor for the determination of 4-MAP through voltammetry techniques. This chitin/f-CNF@GCE has an enhanced surface area and a higher degree of electrocatalytic activity compared to either chitin@GCE or f-CNF@GCE owing to the strong synergistic interfacial interaction between the chitin biopolymer and the f-CNF surface. To the best of our knowledge, there are no previous studies that have reported on the use of innovative chitin/f-CNF nanocomposite for electrochemical 4-MAP detection. The proposed sensor achieved higher sensitivity and selectivity and low-level detection of 4-MAP through reversible redox mechanisms. Moreover, density functional theory (DFT) calculations were performed to investigate the electronic energy landscape and charge-transport pathways of 4-MAP during the electrochemical process. Finally, the proposed sensor was employed to conduct instantaneous analysis of 4-MAP in different water samples such as tap, pond, and river water.

## 2 Experimental Section

### 2.1 Chemicals and Reagents

Chitin powder [(C_8_H_13_O_5_N)_*n*_, 99.9%], 5% acetic acid, and CNFs (conical, D × L, made of 98% carbon, about 100 nm wide and 20–200 μm), 4-MAP (99.9%), HNO_3_ (99%), and H_2_SO_4_ (97%) were sourced from Sigma-Aldrich and Merck companies. A buffer solution for electrochemical impedance spectroscopy (EIS) and interface analysis was prepared using 0.1 M KCl and K_3_FeCN_6_ and K_4_FeCN_6_. Additionally, 0.1 M disodium hydrogen phosphate (Na_2_HPO_4_) and monosodium hydrogen phosphate (NaH_2_PO_4_) were used to prepare a phosphate buffer solution (PBS) at pH 7.0, which served as the electrolyte solution for 4-MAP analysis. The interfering compounds such as caffeic acid (CA), uric acid (UA), dopamine hydrochloride (DAH), ascorbic acid (AA), hydroquinone (HQ), glucose (Glu), 2-nitroaniline (2-NA), mercury (Hg^2+^), and lead (Pb^2+^) used in this work were purchased as analytical grade and utilized without additional purification. The preparation of all stock solutions involved doubly distilled (DD) water. The instruments used are elaborately described in the Supporting Information.

### 2.2 Preparation of Chitin/f-CNF Nanocomposite

To functionalize CNFs with COOH groups, 2g of CNFs was combined with a freshly prepared acid mixture (40 mL of HNO_3_/H_2_SO_4_; 1:3 ratio), stirred until reaching a homogeneous suspension, and later heated to 60 °C under reflux in a round-bottom flask with continuous stirring for 8 h. Afterward, it was cooled to room temperature and poured carefully into 200 mL of water, washed repetitively until it reached pH 7.0, filtered using Whatman No. 1 filter paper, and rinsed with ethanol. Afterward, it was dehydrated at 60 °C for 12 h to collect the f-CNFs. Twenty mg of chitin was mixed with 5% acetic acid and sonicated for 45 min before it was mixed with 5 mg/mL water-dispersed f-CNFs. The mixture was further sonicated for 1 h at 40 kHz frequency with a power of 200 W.^33,34^ The obtained chitin/f-CNF nanocomposites were washed with water and oven-dried overnight at 60 °C; the synthesis procedure is displayed in Scheme 1, and the chemical reactions are expressed in eqs 1–4:

$$\mathrm{CNF}+{\mathrm{HNO}}_{3}/H_{2}{\mathrm{SO}}_{4}\to \mathrm{f‐CNF}\left(\mathrm{-COOH}\right)$$ 1

$$\mathrm{chitin}\left({\mathrm{-NHCOCH}}_{3}\right)+{\mathrm{CH}}_{3}\mathrm{COOH}\to \mathrm{chitin}\left(\mathrm{protonated}{\mathrm{-NH}}^{3+}\right)$$ 2

$$\mathrm{f‐CNF}\left(\mathrm{-COOH}\right)+\mathrm{chitin}\left({\mathrm{-NH}}_{2}\right)\to \mathrm{f‐CNF-CO-NH-chitin}$$ 3

$$\mathrm{f‐CNF}(\mathrm{-OH},\;\mathrm{-COOH})\cdot \cdot \cdot \mathrm{chitin}(\mathrm{-OH},\;{\mathrm{-NH}}_{2}),[\mathrm{physical interaction}]$$ 4

**Scheme 1:**
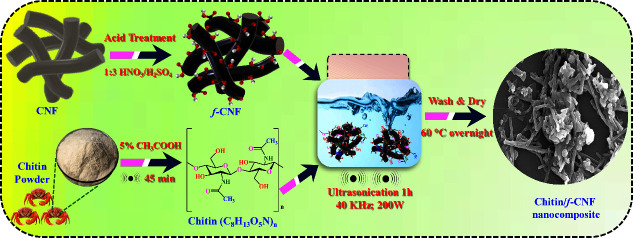
Synthesis of Chitin/f-CNF Nanocomposite

### 2.3 Modification of Chitin/f-CNF@GCE

GCE was polished using an alumina slurry (0.05 μm) to enhance the surface area and sonicated slightly with an ethanol–water mixture for 20s to remove unwanted particles on its surface and finally rinsed with DD water. 6 μL of freshly prepared chitin/f-CNF nanocomposites were drop-casted on the GCE and dehydrated at 60 °C for 10 min. The modified chitin/f-CNF@GCE was employed as a working electrode to detect 4-MAP. For assessment, f-CNF@GCE and chitin@GCE were fabricated separately using the same method. Reproducibility measurements were calibrated using the same polished and nanocomposite-coated electrodes.

## 3 Results and Discussion

### 3.1 Structural Classifications

XRD spectra of chitin, f-CNF, and chitin/f-CNF nanocomposite are presented in Figure 1A. The obtained XRD patterns accurately expose the crystallographic arrangement of the prepared samples, which were refined by PAN analytical X-PERT PRO spectra. Sharp, distinct diffraction peaks of chitin (green) appeared at 2θ = 12.14°, 19.12°, 20.49°, 23.15°, 26.06°, and 38.75°, which correspond to the lattice planes of (020), (110), (120), (130), (013), and (110), respectively. These peak values are consistent with the α-chitin structure; specifically the 2θ values at 19.12° are ascribed to *N*-glucosamine arrangements, and the second main diffraction peak observed at 12.14° are due to *N*-acetyl-d-glucosamine arrangements, further confirming the successful existence of the characteristic crystalline structure of the chitin hydrogel.^35,36^ f-CNF (blue) has a distinct diffraction peak that appeared at 26.6° and a trivial hump at around 44.6° endorsed to (002) and (110) lattice planes, respectively. Results reveal a planar arrangement of the carbon matrix with a specific interlayer spacing of 5.79 Å with the hexagonal carbon structure and space group *P*6̅*m*2 with the JCPDS card no. 00-026-1076 which confirms the formation of characteristic carbon nanofibers. The XRD patterns of the chitin/f-CNF nanocomposite reveal the merged diffraction peaks of chitin and f-CNF, confirming the successful combination. Notably, no significant distortion of the lattice planes re-engineering is obtained, implying the lowest structural disruption. Nevertheless, the improved diffraction peak intensities suggest higher crystallinity in the chitin/f-CNF nanocomposite compared to the individual components, resulting in increased crystallinity, likely due to the synergistic interaction between chitin and f-CNF, which stabilizes their crystalline structures.

**Figure 1:**
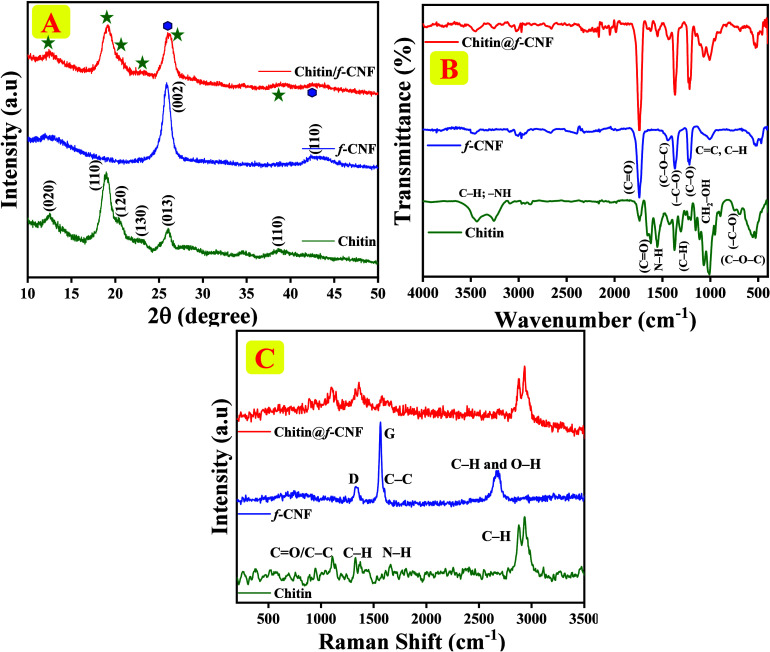
Spectra of chitin (green), f-CNF (blue), and chitin/f-CNF nanocomposite (red). (A) XRD patterns, (B) FT-IR spectra, and (C) Raman spectra.

Moreover, the crystallite size was evaluated using the Debye–Scherrer equation (eq 5):

D=Kλ/βcosθ 5

Here, *D* represents the crystallite size, *K* is Scherrer’s constant, λ denotes the X-ray wavelength, β corresponds to the full width at half-maximum of the diffraction peak, and θ is the diffraction angle. Based on these parameters, the average crystallite size of the chitin/f-CNF nanocomposite was calculated to be 17.35 nm. Overall, the XRD analysis confirms the successful formation of the chitin/f-CNF nanocomposite without any impurities that signals better physical improvement and possibly improved properties for applications in sensing and catalysis.

FT-IR spectra of chitin, f-CNF, and chitin/f-CNF nanocomposite are displayed in Figure 1B. The chitin spectrum exhibits characteristic vibration bands of different glycosidic bands at 523, 566, 1013, and 1066 cm^–1^ which correspond to ν­(C–O–C), (−C–O), CH_2_–OH, and C–OH, respectively. These results confirm the presence of an α-chitin structure in the analyzed materials. Additionally, the bands at 1309 and 1369 cm^–1^ correspond to the symmetric deformation of δ­(C–H) vibrations, further confirming the presence of chitin’s characteristic structure. The main structure of chitin relies on the N–H amide II group and the ν­(C=O) amide I group, as shown through carbonyl stretching and bending vibrations appearing at 1550 and 1625 cm^–1^, respectively. Furthermore, bands at 1742 and 3258 cm^–1^ are linked to *v*(acetoacetate) and ν­(C–H; −NH) symmetrical and asymmetrical stretching modes. Band appearing at 3440 cm^–1^ corresponds to stretching frequencies associated with hydroxyl groups (−OH), also consistent with chitin’s known structural components.^37–39^ For f-CNF, the stretching and bending vibrations of (C=C, C–H, C–O) hydrocarbon functional groups occur at 1007 and 1220 cm^–1^ in f-CNF. The C–O–C, C–O, and C=O stretching vibrations appearing at 1370 and 1742 cm^–1^ confirm the successful functionalization of CNFs, particularly with oxygen-comprising groups that enhance the reactivity and interaction with other materials. A minor peak appearing at 3471 cm^–1^ indicates stretching vibrations in carboxyl groups (−COOH) which enrich the hydrophilicity and electrochemical properties of CNFs.^40–42^ When analyzing the chitin/f-CNF nanocomposite, the obtained FT-IR spectrum shows the overlapping of characteristic bands of both chitin and f-CNF that confirm the successful incorporation of f-CNF into the chitin matrix, with hydrogen bonding and electrostatic interactions enhancing the chitin/f-CNF nanocomposite’s structural and chemical stabilities. The modified composite substances indicate the development of a ground-breaking chitin/f-CNF nanocomposite which exhibits potential applications in biocomposite materials and environmental sensors.

Raman spectra of chitin and f-CNFs as well as the chitin/f-CNF nanocomposite are presented in Figure 1C. For chitin, the Raman spectrum exhibits characteristic peaks in the range of 800–1150 cm^–1^ that resemble the C=O/C–C stretching vibrations. The results confirm C–H deformations between 1309 and 1484 cm^–1^ as well as the amide I functional group at 1657 cm^–1^. C–H stretching modes in chitin produced the C–H stretching vibrations with a primary peak at 2941 cm^–1^ matching the acetyl group in the chitin structure. Spectrum data indicate successful synthesis of chitin according to previous reports.^43,44^ In the f-CNF spectrum, two prominent peaks appear at 1343 and 1555 cm^–1^ that stem from C–C bond scatters showing both sp^2^ and sp^3^ hybridizations of D and G bands. The hump noticed at 2672 cm^–1^ represents C–H and O–H stretching modes, indicating enhanced hydrogen bonding between the structures. This observation replicates the adjustment of CNFs, which is critical for improving their responsiveness and collaboration with other materials. The Raman spectrum of the chitin/f-CNF nanocomposite shows peaks that closely match those of its single components while maintaining clear bands. One specific peak for chitin observed at 2941 cm^–1^ has been slightly shifted in the chitin/f-CNF nanocomposite due to hydrogen bonds between chitin and f-CNF, thus revealing the electronic environment, and likely responsible for the appearance of acidic functional groups in the nanocomposite. These modifications are influenced by potential nanostructural homogeneity, enhanced vibrational modes, and improved structural integrity through synergistic recombination between chitin and f-CNF. These structural improvements can enhance the electrochemical behavior, which has been obtained in consequent electrochemical investigations.^45,46^

### 3.2 Morphological Analysis

FE-SEM and HR-TEM techniques enable us to study individual chitin and f-CNF properties as well as characteristics of the chitin/f-CNF nanocomposite. FE-SEM images at various magnifications shown in Figure 2A,B reveal the distinct morphologies of chitin and f-CNF. Chitin exhibits a stacked, sheet-like flake morphology dependable on its natural form, and f-CNF presents fiber-like networks that establish a connection, as shown in Figure 2C,D. The importance of this fiber-like structure of f-CNF is that it implies large surface area and high connectivity of the material, which is suitable for a composite. Upon oxidative acid treatment, f-CNF surfaces introduced additional functional groups, which led to structural damage of the outer structure through breakdown and crack formation. These cracks and fragmentations in the fibers make them react more and have increased surface area to react with other materials, especially chitin. This is an important addition to enhance the activity of the resulting nanocomposite. The effective integration of chitin into f-CNF networks occurred through ultrasonic processing that was confirmed in Figure 2E,F. The fiber-like arrangement of f-CNF is also seen to be quite compatible with the sheet-like chitin flakes, indicating that there is a favorable interfacial contact leading to a stable nanocomposite. The HR-TEM images (Figure 2G,H) demonstrated the successful combination with a well-mixed structure of fiber-like f-CNF networks closely interconnected with sheet-like chitin flakes creating a regimented and combined nanocomposite. Moreover, the average particle size of the nanocomposite was calculated using ImageJ software and calculated to be 85.9 nm, which was well aligned with the crystallite size calculated by XRD analysis. Additionally, Figure 2I­(a) provides SAED data that confirm the crystalline nature of carbon matrix structures identified through XRD. These obtained data strongly indicate the successful formation of the chitin/f-CNF nanocomposite. The combination of X-ray and EDX information obtained from FE-SEM analysis reveals essential data regarding the form and structure of chitin and f-CNF alone along with their nanocomposite state.

**Figure 2:**
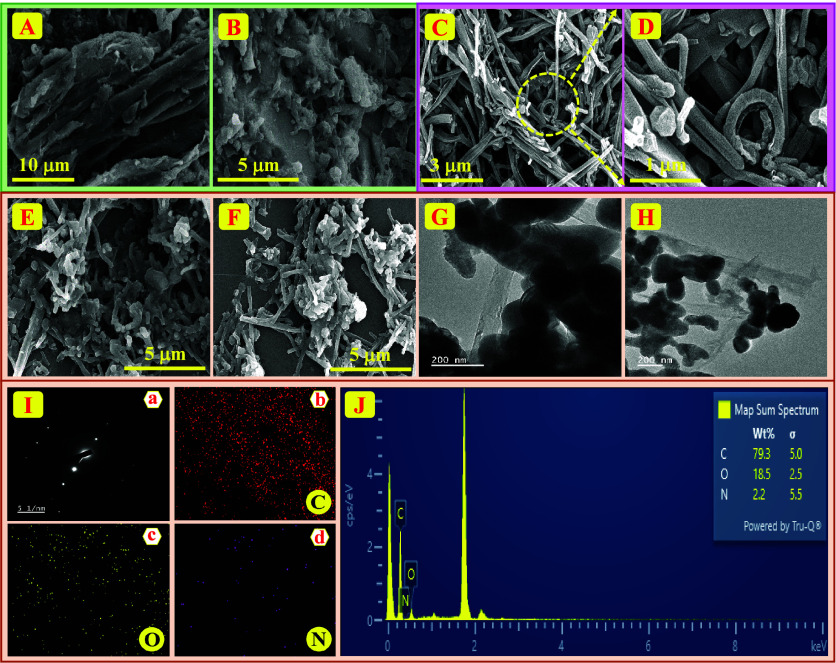
(A, B) FE-SEM images of chitin, (C, D) f-CNF, and (E, F) chitin/f-CNF nanocomposite. (G, H) HR-TEM images. (I) (a) SAED patterns and (b–d) elemental mapping analysis of carbon, oxygen, and nitrogen. (J) EDX patterns of chitin/f-CNF nanocomposite.

We studied the morphology, shape, and interfacial properties of chitin and f-CNF as individual components and their nanocomposite form with FE-SEM and HR-TEM examination. Elemental mapping analysis verified the uniform distribution of all elements which appear in Figure 2I­(b–d). The nanocomposite contains equally dispersed carbon, oxygen, and nitrogen according to elemental mapping analysis, indicating the consistent chitin/f-CNF structure. EDX patterns of the chitin/f-CNF nanocomposite can be observed in Figure 2J. The spectrum shows peaks indicating the presence of carbon (C), oxygen (O), and nitrogen (N), while the resulting data show qualitative along with quantitative features throughout the scanned area. A prominent peak of carbon (C) shows the organic structural component of the material that comes from chitin and controlled-network-formed fibers. A trace peak in the spectrum relates to the oxygen-rich structures such as carboxyl or hydroxyl groups as well as to the nitrogen groups formed, while amine or acetamide groups are a part of chitin. The results conclude that 79.3% carbon, 18.5% oxygen, and 2.2% nitrogen are present. Advanced fields of biosensing and catalysis together with functional composite development show potential due to this materials’ composition characteristics.

### 3.3 EIS Analysis

The electron-transfer kinetics of bare GCE, chitin@GCE, f-CNF@GCE, and chitin/f-CNF@GCE were initially measured by EIS. It was performed in the presence of standard electrolyte solution 5 mM ferro-/ferricyanide [Fe­(CN)_6_]^3–/4–^ with 0.1 M potassium chloride (KCl) solution within the frequency range of 100 Hz to 100 kHz, and the results are displayed in Figure 3A. Randle’s circuit was utilized to simulate an equivalent circuit, incorporating key components such as charge-transfer resistance (*R*_ct_), Warburg impedance (*W*), and solution resistance (*R*_s_) using EIS analysis and is provided in the inset of Figure 3A. The semicircular diameter and charge-transfer resistance decreased in the following order: bare GCE (1300.99 Ω), chitin@GCE (992.59 Ω), f-CNF@GCE (131.08 Ω), and chitin/f-CNF@GCE (18.69 Ω).

**Figure 3:**
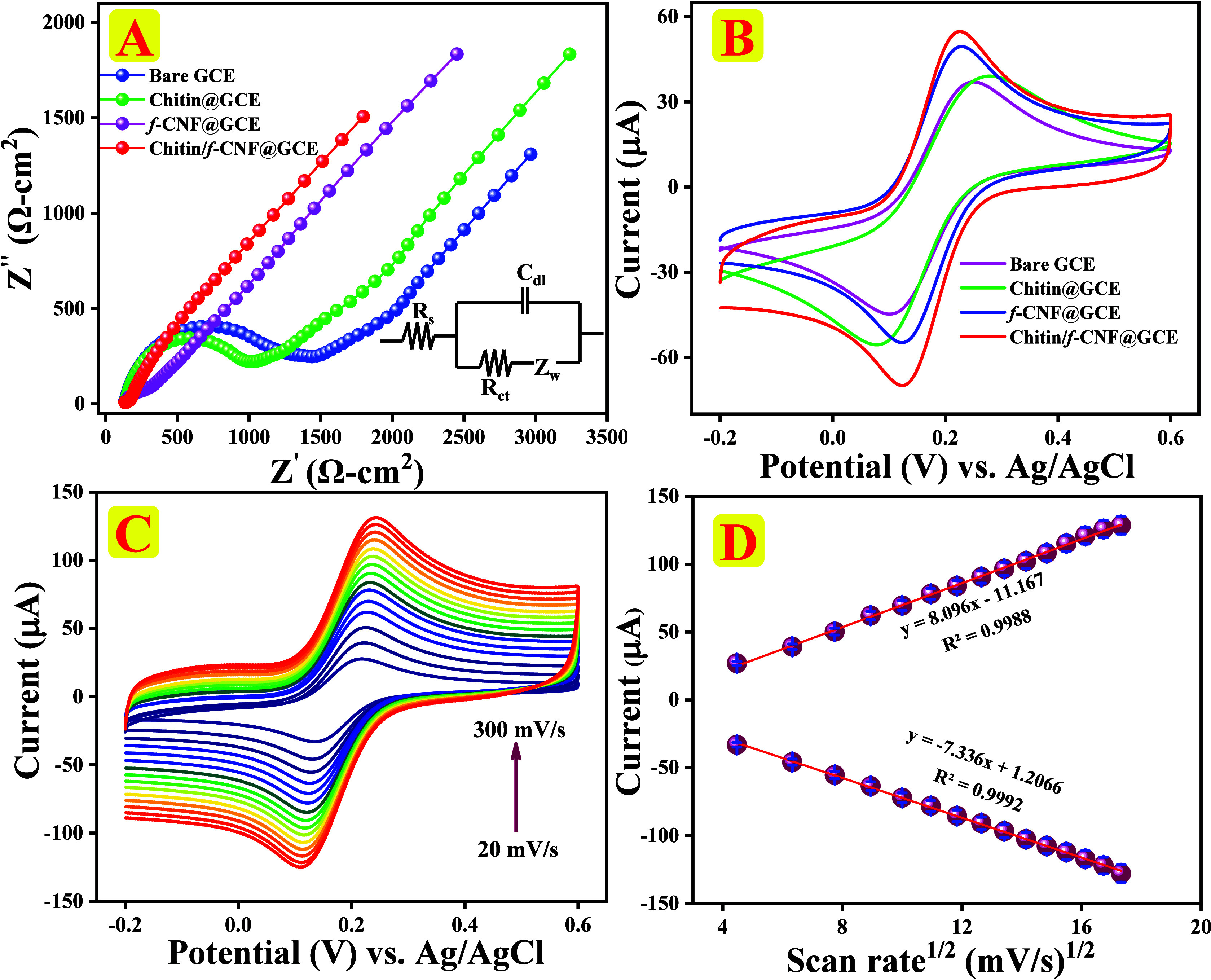
(A) Nyquist plots of bare GCE (blue), chitin@GCE (green), f-CNF@GCE (pink), and chitin/f-CNF@GCE (red) recorded in 0.1 M KCl comprising 5 mM [Fe­(CN_6_)^3–/4–^] solution. (B) CV responses of various modified electrodes at 100 mV/s sweep rate. (C) CV responses of variation in the sweep rate (20–300 mV/s) at chitin/f-CNF@GCE. (D) Consistent linear plot for redox peak current vs square root of sweep rate.

Chitin/f-CNF@GCE exhibited the lowest semicircle and *R*_ct_, which reveals rapid electron transport between the electrolyte and the nanocomposite along with enhanced electrocatalytic performance. The heterogeneous electron-transfer rate constant (*k*_s_) of chitin/f-CNF@GCE was estimated from the charge-transfer resistance (*R*_ct_) values according toeq [Disp-formula eq6]

$$k_{s}=RT/nFAR_{\mathrm{ct}}C$$ 6

where the value of *R* is 8.314 J mol^–1^ K^–1^, the *T* value is 298 K, the f value is 96485 C mol^–1^, *A* is 0.07 cm^2^, *n* denotes the number of electrons transferred, the *C* value is 5 × 10^–6^ mol cm^–3^, and *R*_ct_ is the measured charge-transfer resistance of chitin/f-CNF@GCE which is 18.69 Ω. The *k*_s_ value was calculated to be 3925.6 cm s^–1^.^47^ This high electron-transfer rate suggests that the chitin/f-CNF nanocomposite effectively enhances the conductivity and simplifies fast charge transfer on the electrode surface, corroborating the improved electrochemical performance observed compared to bare and other modified electrodes.

Following EIS analysis, peak-to-peak separation (Δ*E*_p_) and electrochemical active surface area (EASA) of modified and bare electrodes were investigated through cyclic voltammetry (CV), and the results are displayed in Figure 3B. CV responses were recorded in 5 mM [Fe^–^(CN)_6_]^3‑/4–^ comprising 0.1 M KCl solution as an electrolyte at a fixed sweep rate of 100 mV/s. Well-defined redox peaks were observed for bare GCE, chitin@GCE, f-CNF@GCE, and chitin/f-CNF@GCE. Chitin/f-CNF@GCE had a slightly higher peak response compared to all other modified electrodes signifying a superior electrical conductivity of the chitin/f-CNF nanocomposite. It shows the lowest peak-to-peak separation and the maximum peak current response, suggesting that mass diffusion limitation affects the electrode kinetics. Additionally, to understand the diffusion-controlled mechanism of the prepared materials, we performed an influence of sweep rate study by varying the sweep rate (20–200 mV/s) at chitin/f-CNF@GCE, and the results are presented in Figure 3C. It reveals that increasing the sweep rates simultaneously increases the redox peak currents. Moreover, the linear plot for the square root of sweep rate against peak current is displayed in Figure 3D, which clearly illustrates linear dependence of *I*_pa_ (μA) = 8.096 (mV/s)^1/2^ – 11.167 and *I*_pc_ (μA) = −7.336 (mV/s)^1/2^ + 1.2066 with correlation coefficients of *R* ^2^ = 0.9988 (*I*_pa_) and 0.9992 (*I*_pc_), respectively. Furthermore, determination of EASAs of various modified electrodes is the primary means of regulating the electrocatalytic effect, and hence the EASA values have been calculated using Randles–Sevcik eq 7

$$I_{p}=(2.69\times 10^{5})AD^{1/2}n^{3/2}v^{1/2}C$$ 7

Here, *I*_p_, *A*, *D*, *n*, *v*, and *C* stand for the number of electrons, the scanning rate (mV/s), the active surface area (cm^2^), the diffusion coefficient (cm^2^/s), the redox peak current (μA), and the concentration (mol/cm^3^), respectively.^5^ From this equation, EASA values are calculated to be for bare GCE (0.062 cm^2^), chitin@GCE (0.144 cm^2^), f-CNF@GCE (0.317 cm^2^), and chitin/f-CNF@GCE (0.472 cm^2^), revealing that the composite has more excellent active surface area value (0.472 cm^2^). Based on the results, chitin/f-CNF@GCE provides a larger EASA and active sites and well-organized chitin on the surface layer of f-CNFs. This obtained outcome demonstrates that the prepared nanocomposite shows best electrocatalytic behavior for the detection of 4-MAP.

### 3.4 Sensing Performance of 4-MAP at Various Modified Electrodes

The electrochemical detection was recorded using CV technique with 150 μM 4-MAP at bare GCE, chitin@GCE, f-CNF@GCE, and chitin/f-CNF@ in the presence of 0.1 M PBS as an electrolyte solution, which is a physiologically relevant, stable pH environment essential for reliable electrochemical sensing. The sweep rate was 100 mV/s, the potential range was −0.3 to 0.4 V, and the obtained CV curves are displayed in Figure 4A. The redox reactions of 4-MAP at all electrodes involve two electrons (2e^–^) and two protons (2H^+^) reversible reactions from 4-(methylamino) phenol hydrogen sulfate to 4-(methylamino) cyclohexane-2,5-diene-1-one hydrogen sulfate,^6^ as displayed in Figure 4C. Anodic (*E*_pa_) and cathodic (*E*_pc_) peak potentials along with redox peak currents (*I*_pa/pc_) were noted for each modification on the electrode surface and bare GCE. Compared to bare GCE [*I*_pa_ = 9.744 μA, *I*_pc_ = −9.108 μA at a potential of Δ*E*_p_ (0.042 V)], chitin@GCE displayed a higher redox peak current [*I*_pa_ = 14.94 μA, *I*_pc_ = −12.60 μA and Δ*E*_p_ (0.042 V)] which reveals the electrocatalytic role of chitin as a modifier film. On the other hand, compared to chitin@GCE, f-CNF@GCE had superior catalytic response that exhibited optimistic alterations in the redox potential and a smaller peak-to-peak separation (Δ*E*_p_). Specifically, *I*_pa_ for f-CNF@GCE was 21.02 μA, *I*_pc_ was −53.42 μA, and Δ*E*_p_ = 0.031 V, which can be ascribed to enlarged active sites and strong interaction between 4-MAP and f-CNF@GCE. Chitin/f-CNF@GCE demonstrated the most enhanced redox performance for 4-MAP, with *I*_pa_ = 62.18 μA, *I*_pc_ = −65.71 μA, and Δ*E*_p_ = 0.053 V, surpassing that of other modified electrodes. Redox peak responses upsurge in the following order: bare GCE < chitin@GCE < f-CNF@GCE < chitin/f-CNF@GCE. Chitin/f-CNF@GCE had the lowest peak-to-peak separation for the redox cycle of 4-MAP. Histogram responses of different modified electrodes are presented in Figure S1A.

**Figure 4:**
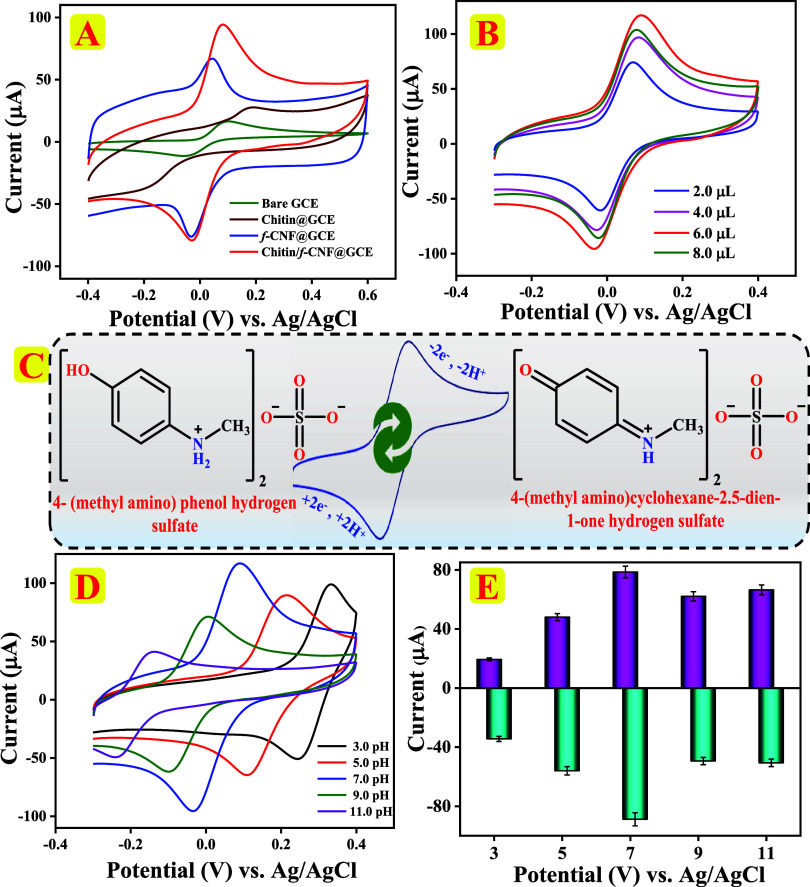
(A) CV responses of bare GCE (green), chitin@GCE (brown), f-CNF@GCE (blue), and chitin/f-CNF@GCE (red) in 150 μM 4-MAP using 0.1 M PBS (pH 7.0) electrolyte at 100 mV/s sweep rate. (B) Redox peak CV curves of 4-MAP on GCE loaded with diverse amounts of 2.0–8.0 μL of the chitin/f-CNF nanocomposite in the presence of 200 μM 4-MAP. (C) Electrochemical mechanism of 4-MAP at chitin/f-CNF@GCE. (D) CV responses of 200 μM 4-MAP on chitin/f-CNF@GCE in 0.1 M PBS with different pH values 3.0–11.0 and (E) corresponding bar diagram.

#### 3.4.1 Effect of Chitin/f-CNF Nanocomposite Loading Amount

The impact of chitin/f-CNF nanocomposite loading amount on the GCE surface was assessed using the CV technique, which significantly influenced the sensitivity and current response of modified electrodes. We evaluated different loading levels of 2.0, 4.0, 6.0, and 8.0 μL in the presence of 200 μM 4-MAP in 0.1 M PBS (pH 7.0) at a 100 mV/s sweep rate, and the obtained results are displayed in Figure 4B. Redox peak currents increased with increasing loading amounts from 2.0 to 6.0 μL and decreased with a further increase to 8.0 μL. The corresponding bar graph is illustrated in Figure S1B.

CV results indicate that the highest current response of 78.49 μA was attained with 6.0 μL of chitin/f-CNF nanocomposite suspension, which was thus identified as the optimal loading amount for electrocatalytic detection of 4-MAP, and it is used for all further electrochemical experiments.

#### 3.4.2 Effect of pH

To examine the redox behavior of 200 μM 4-MAP, CV was performed at a sweep rate of 100 mV/s in 0.1 M PBS in the pH range of 3.0–11.0, and CV responses are presented in Figure 4D. The corresponding bar chart displaying discrepancy in the peak current vs pH is presented in Figure 4E. As the pH of the electrolyte solution increased, the redox peak potential shifted to more negative values, while the redox peak current initially enlarged from pH 3.0 to 7.0 and subsequently diminished from pH 7.0 to 11.0. These findings suggest that the chitin/f-CNF@GCE electrode maintained an equal number of electron and proton transfer, with the utmost peak current at pH 7.0 (*I*_pa_ = 64.18 μA, *I*_pc_ = −67.71 μA, and Δ*Ε*_p_ = 0.053 V). A clear linear relationship between the peak potential of 4-MAP and the electrolyte pH is shown in Figure S1C with linear regressions *E*_pa_ = −0.0593pH + 0.4136 and *E*_pc_ = −0.0576pH + 0.5025 and correlation coefficients *R* ^2^ = 0.9979 (*E*_pa_) and *R* ^2^ = 0.9964 (*E*_pc_). The obtained shifts conform to the Nernst eq 8.

$$dE_{p}/d_{\mathrm{pH}}=(2.303mRT)/(nF)$$ 8

From eq 8substituted with the slope of *E*_p_ vs pH plot, *m* is estimated to be 2. It reveals that the electrochemical process at chitin/f-CNF@GCE on 4-MAP is a two-electron, two-proton transfer process.^48^ Based on these outcomes, all further electrochemical studies were performed at electrolyte pH 7.0.

#### 3.4.3 DFT Studies

DFT calculations were performed to investigate and study the electronic properties of 4-MAP. B3LYP functional with the 6-311G­(d,p) basis set was used to optimize the 4-MAP molecule and to investigate whether the optimized molecule is at true local minimum. The optimized molecular geometry revealed a stable 4-MAP molecule with no imaginary frequencies, confirming that the structure is at a true local minimum. Figure 5A displays the optimized structure of 4-MAP. The electrostatic potential map (Figure 5B) was generated to visualize the charge distribution across the 4-MAP molecule, showcasing regions of negative potential near electronegative atoms (oxygen and nitrogen).

**Figure 5:**
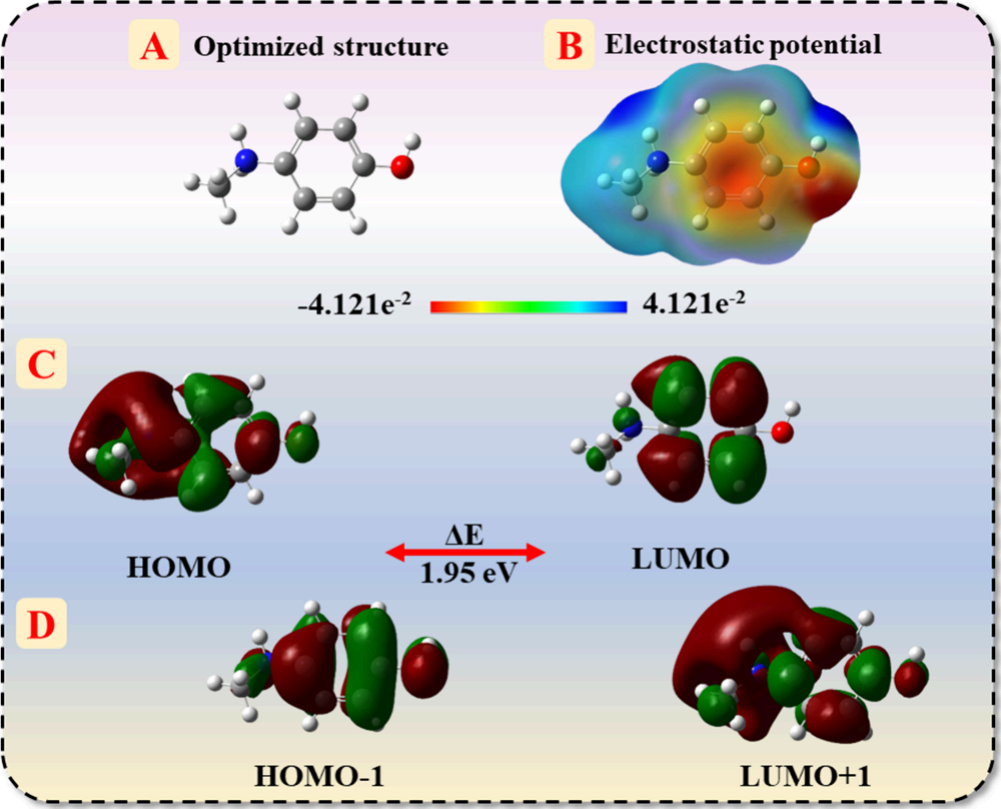
Theoretical DFT analysis of 4-MAP illustrating (A) optimized molecular structure, (B) molecular electrostatic potential map, and (C, D) frontier molecular orbitals (HOMO, LUMO, HOMO–1, and LUMO+1).

This region may serve as active sites for intermolecular interactions or hydrogen bonding between 4-MAP and chitin/f-CNF@GCE. Furthermore, frontier molecular orbitals, namely, the highest occupied molecular orbital (HOMO) and the lowest unoccupied molecular orbital (LUMO) of 4-MAP were analyzed to understand its molecular reactivity.^49,50^ The HOMO represents the ability of 4-MAP to donate electrons, while the LUMO signifies the ability to accept electrons. HOMO is predominantly localized over the amino and hydroxyl groups of 4-MAP, while LUMO is mainly distributed over the aromatic ring as displayed in Figure 5C with a band gap of 1.95 eV. The HOMO–1 shows significant delocalization over the aromatic ring, while LUMO+1 extends to other regions of the molecule (Figure 5D). Some global reactivity parameters have been calculated usingeqs [Disp-formula eq9]–[Disp-formula eq15]

$$\Delta E=E_{\mathrm{LUMO}}-E_{\mathrm{HOMO}}$$ 9

$$\mathrm{IP}=-E_{\mathrm{HOMO}}$$ 10

$$\mathrm{EA}=-E_{\mathrm{LUMO}}$$ 11

$$\eta =E_{\mathrm{LUMO}}-E_{\mathrm{HOMO}}/2$$ 12

$$\mu =-E_{\mathrm{HOMO}}+E_{\mathrm{LUMO}}/2$$ 13

χ=−μ 14

$$\omega =\mu^{2}/2\eta$$ 15

where Δ*E* is the energy gap, *E*_LUMO_ is the energy of LUMO, *E*_HOMO_ is the energy of HOMO, IP is the ionization potential, EA is the electron affinity, η is the chemical hardness, μ is the chemical potential, χ is electronegativity, and ω is the electrophilicity index. The calculated global parameters have been listed in Supporting Information Table S1. This detailed orbital and electrostatic potential analysis supports the experimentally observed redox mechanism and provides some theoretical basis, highlighting the role of electron density in electron and proton transfer between chitin/f-CNF@GCE and 4-MAP interface.

#### 3.4.4 Effect of 4-MAP Quantity

The redox performance of chitin/f-CNF@GCE was evaluated by CV in 0.1 M PBS (pH 7.0) at 100 mV/s sweep rate with altered concentrations of 4-MAP extending from 20 to 400 μM, and the obtained results are shown in Figure 6A. The results show that the redox peak response increases gradually with increasing 4-MAP concentrations, which supports the outstanding electrocatalytic behavior that was controlled by the mass transfer of 4-MAP to the chitin/f-CNF@GCE surface. The relationship flanked by 4-MAP concentrations and redox peak currents was quantified through the following regression values *I*_pa_ (μA) = 0.3809 (μM) + 2.5575; *I*_pc_ (μA) = −0.3946 (μM) – 5.2164 with respective correlation coefficients *R* ^2^ = 0.9939 (*I*_pa_) and *R* ^2^ = 0.9915 (*I*_pc_) respectively. The redox pair’s correlation coefficient was near 1, indicating the superior redox characteristics of 4-MAP, as displayed in Figure 6B, which evidenced that chitin/f-CNF@GCE follows first-order kinetics toward the electrochemical detection of 4-MAP.

**Figure 6:**
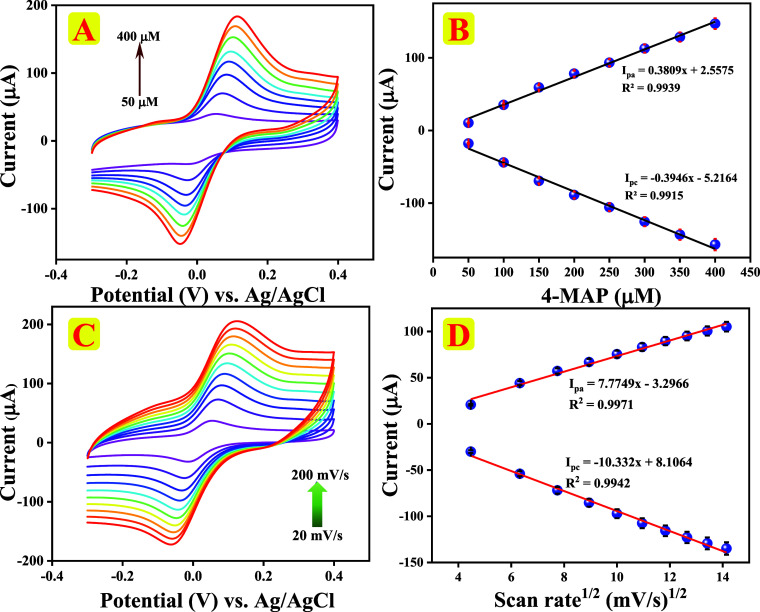
(A, B) CV responses of chitin/f-CNF@GCE at different 4-MAP concentrations (50–400 μM) in 0.1 M PBS (pH, 7.0) at 100 mV/s sweep rate and the connection plot between the redox peak current and 4-MAP concentration. (C, D) CV curves at different sweep rates (20–200 mV/s) in 0.1 M PBS with 200 μM 4-MAP and the equivalent calibration plot of redox peak current vs square root of sweep rate.

#### 3.4.5 Effect of Sweep Rate

Effect of sweep rate on the sensing performance of chitin/f-CNF@GCE was systematically examined using CV in 0.1 M PBS (pH 7.0) with 200 μM 4-MAP. Figure 6C illustrates the association between the redox peak current and sweep rate (*v*), and a linear increment in redox peak response was observed as the sweep rate was increased from 20 to 200 mV/s. Figure S1D highlights the linear correlation and regression equations as follows: *I*_pa_ (μA) = 0.4301 mV/s + 26.442; *I*_pc_ (μA) = −0.5538 mV/s – 33.986 with correlation coefficients of *R* ^2^ = 0.9385 (*I*_pa_) and *R* ^2^ = 0.9486 (*I*_pc_). The above observations were very consistent with the calibration curve displayed in Figure 6D, which shows extraordinary linearity between the square root of sweep rate (mV/s)^1/2^ and the redox peak current (μA). *I*_pa_ (μA) = 7.7749 mV/s – 3.2966; *I*_pc_ (μA) = −10.332 mV/s + 8.1064, with relationship coefficients of *R* ^2^ = 0.9971 (*I*_pa_) and 0.9942 (*I*_pc_) respectively.

The improved correlation and linearity propose that the redox process is governed by diffusion kinetics rather than a surface-controlled process. Figure S1E presents the Laviron plot, illustrating a linear relationship between the anodic peak potential (*E*_pa_) and the logarithm of anodic peak current (log *I*_pa_) at chitin/f-CNF@GCE with eq 16.

$$E_{\mathrm{pa}}=E^{0}+2.303RT/(1-\alpha )nF\;\log\;v$$ 16

Figure S1F depicts the linear relationship between the peak potentials and the logarithm of sweep rates of the electrode potential derived within the range of 20–200 mV/s, which revealed that while increasing the sweep rate, there is a slight shift in peak potentials toward less positive values with linearity. Corresponding linear regression equations are *E*_pa_ = 0.0696*x* – 0.0441 with a correlation coefficient of *R* ^2^ = 0.9679. For a reversible electron-transfer process, according to Laviron model, the peak potential (*E*_p_) is related to the sweep rate (*v*) as described byeq [Disp-formula eq17]

$$\log\;k_{s}=\alpha \;\log(1-\alpha )+(1-\alpha )\log\;\alpha -\log(RT/nFv)-\alpha (1-\alpha )nF\Delta E_{p}/2.303RT$$ 17

where *k*_s_ is the standard heterogeneous electron transfer rate constant, α is the charge-transfer coefficient, *n* is the number of electrons transferred (*n* = 2), *v* is the sweeping rate (100 mV/s), and Δ*E*_p_ represents peak-to-peak separation (Δ*E*_p_ = 0.060 V). Other constants remain the same as aforesaid. The Δ*E*_p_ value was obtained by inducing peak separation at a sweep rate of 100 mV/s. Electron-transport coefficient (α) was calculated from the slope of linear plots with help of relationship (eq 18)­

$$\log(k_{a}/k_{c})=\log(\alpha /1-\alpha )$$ 18

From the calculations, the electron-transfer coefficient (α) for the redox behavior of 4-MAP was determined to be 0.425, indicating nearly equal electron-transfer kinetics between chitin/f-CNF@GCE and the redox probe. Furthermore, the number of electrons involved in the redox process was confirmed to be two, and the usual heterogeneous electron-transfer rate constant (*k*_s_) for chitin/f-CNF@GCE system was calculated to be 1.88 s^–1^.^51^

#### 3.4.6 Determination of 4-MAP

Differential pulsed voltammetry (DPV) is a more sensitive and precise technique than CV. In this study, we calibrated DPV responses of chitin/f-CNF@GCE in the range of various concentrations of 4-MAP in 0.1 M PBS (pH 7.0) as an electrolyte, and the resulting data are presented in Figure 7A. The analysis was conducted under ideal conditions using 0.1 M PBS with different additions of 4-MAP increasing from 0.01 to 747.19 μM within the calibration potential from −0.4 to 0.4 V. As 4-MAP was added, the oxidation peak current amplified linearly. In contrast, the highest current was observed during anodic oxidation. The DPV peak signals consistently increased with the concentration of 4-MAP, and its corresponding calibration plot is represented in Figure 7B with two linear graphs of anodic oxidation current increment at 4-MAP concentration spanning from 0.01 to 9.19 μM and 14.19 to 747.19 μM, respectively.

**Figure 7:**
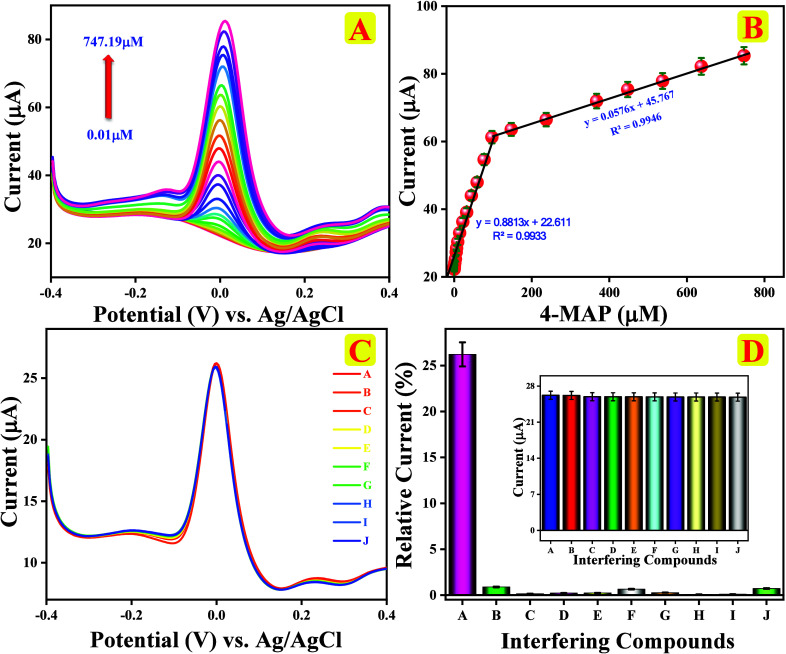
(A) DPV responses of chitin/f-CNF@GCE at 4-MAP concentrations (0.01–747.19 μM) in 0.1 M PBS (pH 7.0), (B) linear plot for oxidation peak current vs 4-MAP concentration, (C) anti-interference analysis, and (D) relative error % against interfering compounds with 4-MAP (inset: peak current vs interfering compounds).

The correlation coefficient (*R* ^2^) was found to be 0.9933 for the lowermost concentration range and 0.9946 for the uppermost range, emphasizing that chitin/f-CNF@GCE is quantifiable for 4-MAP detection. Additionally, the acquired slope value was used to evaluate the sensitivity as well as the quantification limit (LOQ) and detection limit (LOD), using eqs 19–21.

LOD=3×Sb/S 19

LOQ=10×Sb/S 20

sensitivity=slope/EASA 21

The standard deviation of blank is denoted as Sb, and the slope (*S*) is derived from the linear regression equation. Upon analyzing the above equations, the LOD, LOQ, and sensitivity were determined to be 4.2 nM, 14.15 nM, and 1.867 μA μM^–1^ cm^–2^, respectively.

Table 1 compares the investigative characteristics of the improved electrode with earlier issued outcomes for the determination of 4-MAP across various improved electrodes, which revealed that compared to other modified electrodes, chitin/f-CNF@GCE provides a broad linear range and the lowest LOD.

**Table 1:** Assessment of Previous Research Works for 4-MAP Detection with Chitin/f-CNF@GCE

modified electrode	technique	linear range (μM)	LOD (μM)	ref
FeCo@NC/GCE	DPV	0.08–450	0.024	^2^
LSO@f-HNT/GCE	DPV	0.01–480	0.0021	^5^
CaSnO_3_/GCE	DPV	0.01–157	0.003	^6^
MnMoO_4_/SPCE	DPV	0.01–375.6	0.98	^10^
CoMn_2_O_4_@rGO/SPCE	DPV	0.01–137.65	0.050	^14^
ZnO/2D-BCN/GCE	DPV	0.039–1617	0.0086	^51^
Ba-CuO@CB/GCE	DPV	0.01–1000	0.30	^52^
CoMoSe_2_/GO/GCE	DPV	0.04–123	0.09	^53^
Sm_2_(MoO_4_)_3_/CPE	DPV	0.1–300	0.047	^54^
chitin/f-CNF@GCE	DPV	0.01–747.19	0.0042	this work

#### 3.4.7 Selectivity Analysis

The very important consideration while addressing the preciseness of the offered sensor using the DPV approach is selectivity, particularly concerning chemicals that may interfere with the target analyte. In this study, we examined the selectivity of the chitin/f-CNF@GCE sensor in the presence of several substances as displayed in Figure 7C, including 4-MAP (100 μM), alongside with 5-fold additional interfering compounds such as CA (500 μM), UA (500 μM), DAH (500 μM), AA (500 μM), HQ (500 μM), Glu (500 μM), and 2-NA (500 μM). We also examined the effects of cations such as mercury (Hg^2+^; 500 μM) and lead (Pb^2+^; 500 μM). Figure 7D displays the relative error plot of interfering chemicals relative to the anodic peak response of 4-MAP. The chitin/f-CNF@GCE sensor demonstrated remarkable selectivity to determine 4-MAP in complex model environments, evidenced by the minimal impact of interfering compounds. The presence of multiple coexisting chemicals in actual water samples can typically hinder effective detection. Hence, the observed results reveal that the proposed chitin/f-CNF@GCE sensor probe is highly selective toward electrochemical detection of 4-MAP.

#### 3.4.8 Practical Applications of the Proposed Sensor

To obtain the efficiency of the electrochemical sensor utilizing CV and DPV approach, we investigated its reproducibility, repeatability, and stability analysis using 4-MAP at chitin/f-CNF@GCE. The reproducibility examination was accomplished using five various modified GCEs, as shown in Figure 8A, with 0.1 M PBS (pH 7.0) containing 4-MAP (50 μM). The anodic peak current and its relative standard deviation (RSD) were examined, and the anodic currents for five distinct electrodes remained consistent, with no variation. The bar diagram of peak current responses from Figure 8B indicates excellent reproducibility minimal signal variation which demonstrates the improved reproducibility of chitin/f-CNF@GCE. Repeatability was assessed using the DPV method by five repetitive analyses in the presence of 4-MAP (50 μM). The anodic peak current remained stable from first to last measurement, and the corresponding bar chart is illustrated in Figure 8C,D.

**Figure 8:**
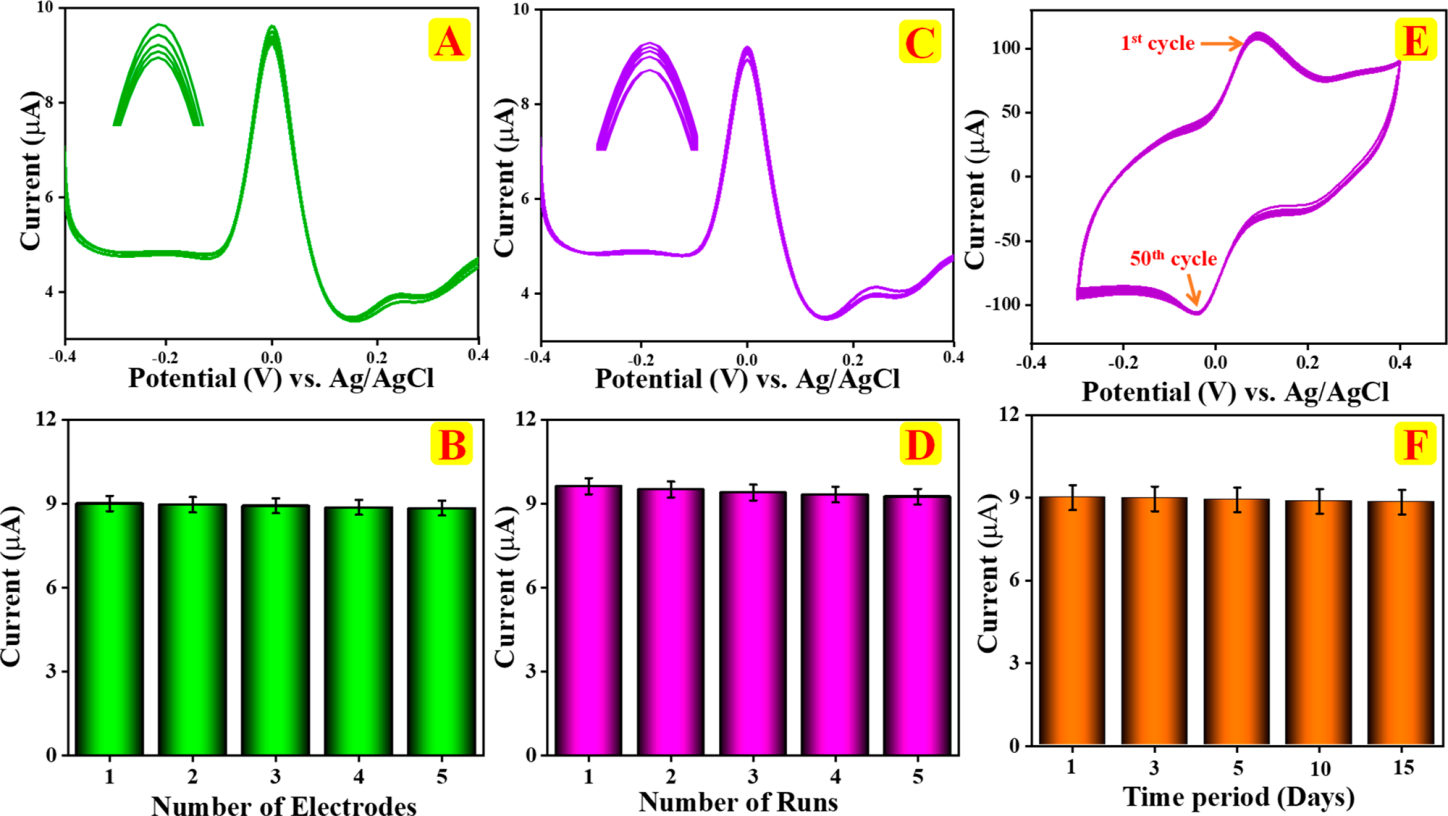
DPV response of (A) reproducibility and (B) corresponding bar diagram. (C) Repeatability and (D) corresponding bar diagram. (E) CV response of cyclic stability showing 50 cycles. (F) DPV response of reusability bar diagram depicting the long-term operational stability of the sensor, performed in the presence 0.1 M PBS (pH 7.0).

Additionally, cyclic stability of the proposed sensor was evaluated by CV over 50 successive cycles in the presence of 4-MAP (200 μM) in 0.1 M PBS (pH 7.0) at a sweep rate of 100 mV/s, as illustrated in Figure 8E. CV data revealed that the redox peak current only declined by less than 5% throughout 50 cycles, indicating that the proposed chitin/f-CNF@GCE sensor exhibits exceptional stability. Figure 8F shows the bar diagram illustrating the reusability of the chitin/f-CNF@GCE sensor with 0.1 M PBS (pH 7.0) containing 4-MAP. Each bar represents the oxidation peak current measured at different intervals. It confirms the durability and reusability of the chitin/f-CNF@GCE electrode during continuous electrochemical operation.

#### 3.4.9 Real-World Environmental Sample Preparation and Analysis

Water samples from river, pond, and tap sources in New Taipei City were collected in precleaned bottles washed with the same water before collection to prevent contamination. We transported the samples, stored them at 4 °C, and normalized the temperature to maintain integrity. The samples were centrifugated at 6000 rpm for 30 min to eliminate impurities and filtered using Whatman No. 42 paper. DPV was used to validate 4-MAP in the prepared water samples in the chitin/f-CNF@GCE sensor and determined through standard addition method. The responses for 4-MAP spiked in tap, pond, and river water were recorded and are displayed in Figure 9A–C. The calibration plots shown in Figure 9D–F demonstrated outstanding linearity for 4-MAP detection in chitin/f-CNF@GCE across various real water samples, with correlation coefficients (*R* ^2^) of 0.9977 (tap water), 0.9930 (pond water), and 0.9994 (river water). The results suggest that chitin/f-CNF@GCE is an effective electrocatalyst, enhancing the detection of 4-MAP in real samples from industries. The sensor’s capability to detect 4-MAP in water sediments and their recovery results are illustrated in Table 2.

**Figure 9:**
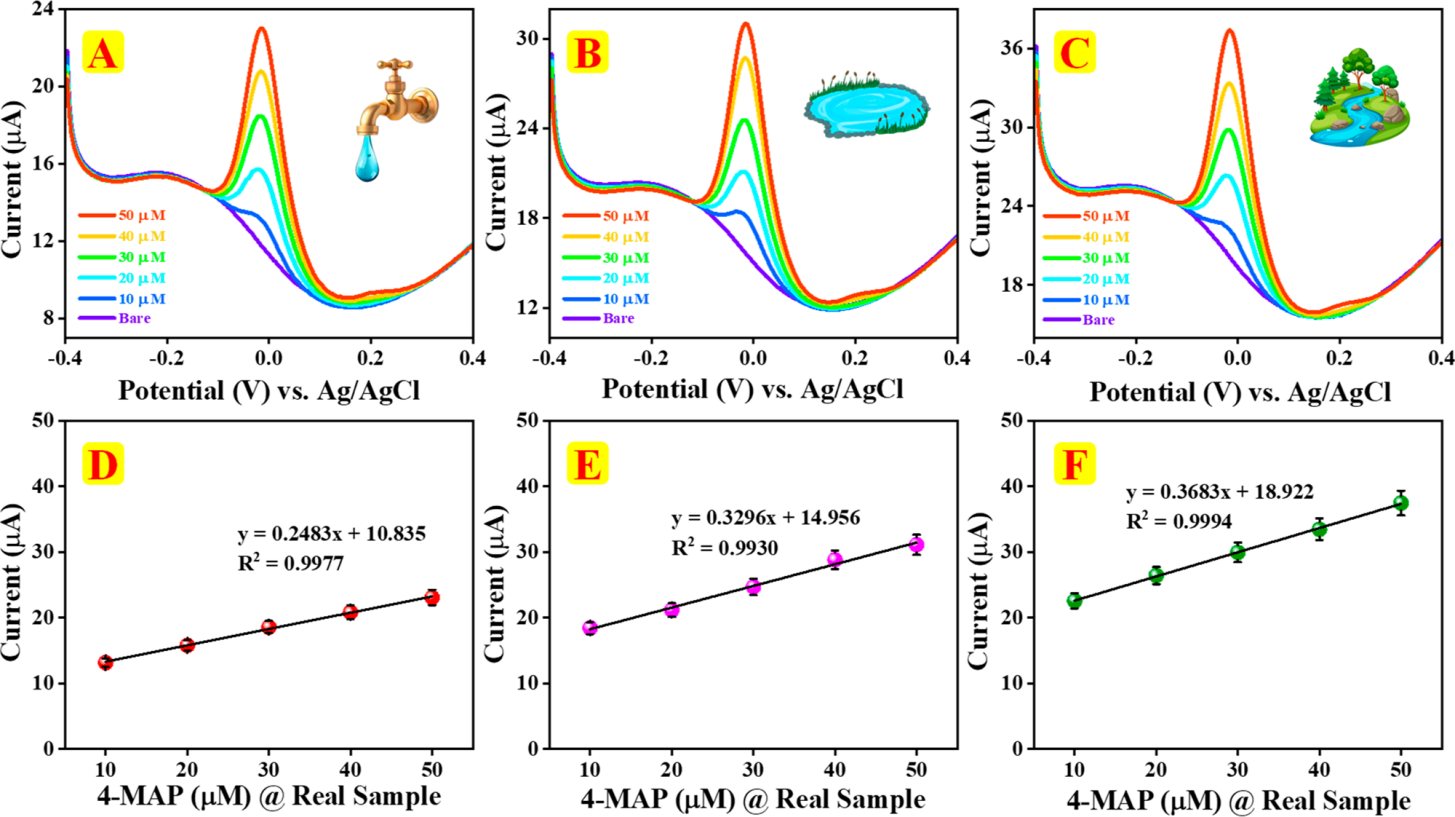
(A–C) DPV signals of chitin/f-CNF@GCE for 4-MAP in various (tap, pond, and river) water samples. (D–F) Calibration curves showing the linear response of chitin/f-CNF@GCE for 4-MAP in various (tap, pond, and river) water samples.

**Table 2:** Detection Response of 4-MAP in Various Real Samples with Recovery Results

sample	added (μM)	found (μM)	RSD (%)	recovery (%)
tap water	10	9.87	2.21	98.72
	20	19.29	1.31	96.45
	30	29.30	2.05	97.77
	40	39.55	1.55	98.87
	50	48.94	3.47	97.88
pond water	10	9.95	1.56	99.51
	20	19.62	0.77	98.10
	30	29.84	1.32	99.46
	40	39.67	3.54	99.17
	50	49.47	2.44	98.94
river water	10	9.79	0.69	97.91
	20	19.72	0.86	98.61
	30	29.66	1.39	98.86
	40	39.88	2.35	99.70
	50	49.79	2.67	99.58

## 4 Conclusions

In conclusion, we have successfully synthesized a chitin/f-CNF nanocomposite through a sustainable ultrasonication-assisted process and used it as an outstanding electrocatalyst for the detection of 4-MAP in environmental samples. The prepared nanocomposite was well characterized by XRD, Raman spectroscopy, FT-IR, FE-SEM, HR-TEM, EDX, and elemental mapping analyses, which confirmed the crystalline nature of the composite with chitin forming stacked sheet-like flakes synergistically interconnected with fiber-like networks of f-CNF and the presence of necessary elements. Plentiful hydroxyl and carboxyl functional groups on f-CNF simplified the robust binding with chitin by surface complexation through synergistic interactions. Afterward, the chitin/f-CNF nanocomposite modified over the GCE surface achieved a low LOD of 4.2 nM, a broad linear range of 0.01–747.19 μM, as well as LOQ of 14.15 nM. Notably, the sensor showed high selectivity toward different interfering species and a sensitivity of 1.867 μA μM^–1^ cm^–2^ with reproducibility, repeatability, and long-term stability over 15 days. DFT revealed HOMO localization on amino and hydroxyl groups (electron-donating sites) and LUMO distribution over the aromatic ring (electron-accepting regions) with a band gap of 1.95 eV. Moreover, the sensor is reliably demonstrated in real environmental water samples and produces acceptable recovery rates. Collectively, these results highlight its strong potential for practical deployment in 4-MAP detection within real matrices and indicate promising prospects for broader analytical applications. The difficulties of CNF aggregation, low conductivity of chitin, and matrix interferences still exist, and the synthesis of CNFs and functionalization need to be enhanced. Despite this, the sensitivity, low cost, environmental friendliness, and scalability of the sensor show promising potential as a biocompatible platform for portable, next-generation devices in a sustainable 4-MAP monitoring platform.

## Supplementary Material



## Data Availability

Data used are available throughout the manuscript text.

## Notes

Instrumental analysis; bar graphs of various electrodes, bare GCE, chitin@GCE, f-CNF@GCE, and chitin/f-CNF@GCE in 150 μM 4-MAP recorded in 0.1 M PBS (pH 7.0) electrolyte at a sweep rate of 100 mV/s; bar graph of diverse amounts of chitin/f-CNF nanocomposite 2.0–8.0 μL; *I*_p_ and *E*_p_ vs pH for chitin/f-CNF@GCE; redox peak current vs sweep rate; linear relationship between the anodic peak potential (*E*_p__a_) and the logarithm of the anodic peak current (log *I*_p__a_) for chitin/f-CNF@GCE; *E*_p_ vs. log sweep rate for chitin/f-CNF@GCE; and calculated energy values and reactivity parameters of Metol (PDF)
