# Supplementary material for: Chitin-Functionalized Carbon Nanofiber-Based Electrochemical Sensor for Rapid and Sensitive Detection of 4‑Methylaminophenol in Aquatic Ecosystems
Source: ACS Omega. 2025 Oct 14;10(42):50530–44. doi: 10.1021/acsomega.5c08044 (PMC12572986; doi:10.1021/acsomega.5c08044)
Supplement: Supplementary file 1 [file ao5c08044_si_001.pdf]

Supporting Information

Chitin/Functionalized Carbon Nanofiber Based Electrochemical sensor for Rapid and Sensitive Detection of 4-Methylaminophenol in Aquatic Ecosystems

Gomathisankar Palavesanarayanan[a], Balamurugan Arumugam[a,d], Rajendran Surya[b,c], Krishnan Venkatesh[a,e], Subramanian Sakthinathan[b,c*], Te-Wei Chiu[b,c*], Sayee Kannan Ramaraj[a*]

[a]PG & Research Department of Chemistry, Thiagarajar College, Madurai, 625009, Tamil Nadu, India.

[b]Department of Materials and Mineral Resources Engineering, National Taipei University of Technology, No.1, Section 3, Chung-Hsiao East Road, Taipei, 106, Taiwan.

[c]Institute of Materials Science and Engineering, National Taipei University of Technology, No. 1, Section Chung-Hsiao East Road, Taipei, 106, Taiwan.

[d]Department of Chemistry, National Sun Yat-sen University, No. 70, Lien-hai Road, Kaohsiung, Taiwan, 804201.

[e]Department of Chemistry, Kyungpook National University, South Korea.

*Corresponding Authors E-mail

Prof. Subramanian Sakthinathan – sakthinathan1988@gmail.com

Prof. Te-Wei Chiu – tewei@ntut.edu.tw

Prof. Sayee Kannan Ramaraj – sayeekannanramaraj@gmail.com

Total number of pages: 4

Total number of figures: 1

Total number of Tables: 1

1. Instrumental analysis

X-ray diffraction analysis (XRD) was employed to study the sample configuration (Bruker XRD, D2 Phaser, Billerica, MA, USA, $\lambda = 1.540$ Å), and PAN analytical X-PERT PRO spectra were utilized to evaluate the data for preparation Delta DC200 ultrasonicator (40KHz; 200w) was used. Nanocomposite analysis over Fourier transform infrared spectroscopy (JASCO FT-IR-4600) showed evidence of functional groups together with compounds. The Witec Inc. system (Ulm, Germany) equipped with a 532 nm Nd-YAG green laser operated at 50mW measured Raman spectroscopy data over 10 seconds per accumulation time to examine vibrational properties. Morphological analysis used a 200 kHz Field Emission Scanning Electron Microscope (FE–SEM, FEI Quanta FEG 200) and High-Resolution Transmission Electron Microscope (HR–TEM) operated at H-7600, Hitachi, Tokyo, Japan. The electrochemical analyses were performed using CHI1211B potentiostat (CH Instruments, Inc., Austin, TX, USA) connected with the three-electrode cell arrangement comprising of an Ag/AgCl reference electrode, a GCE (surface area 0.072 cm^2) working electrode, and a Pt wire counter electrode.

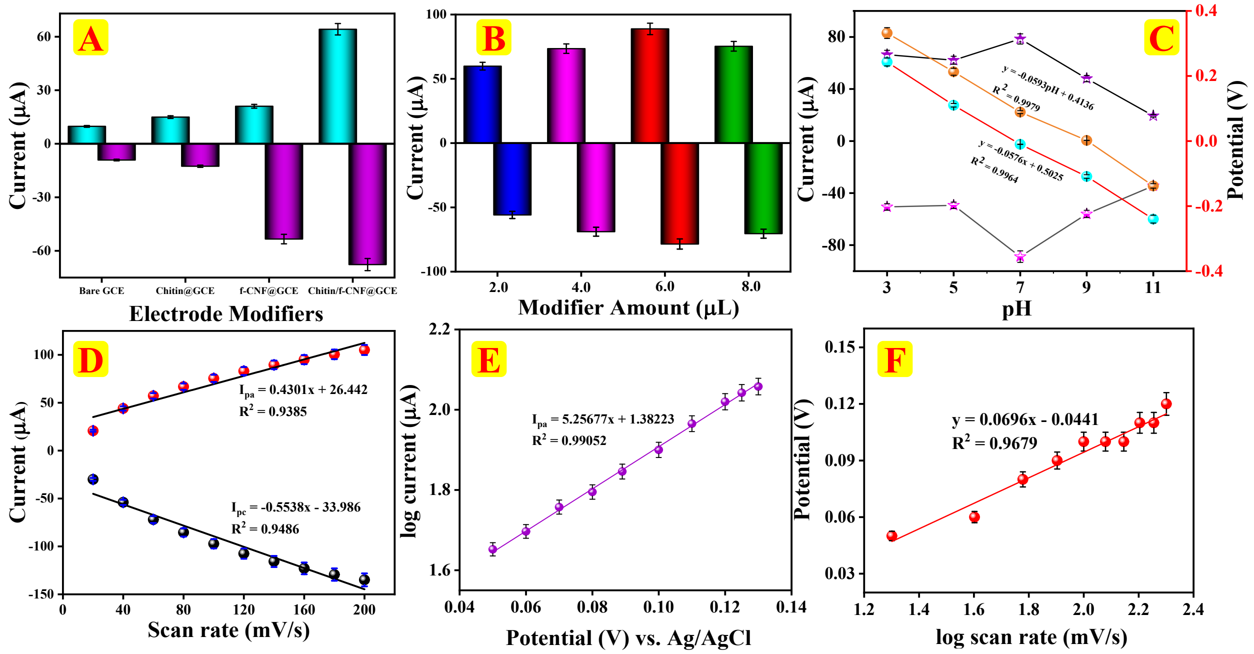

Figure S1. **[A]** Bar graphs of various electrodes bare GCE, Chitin@GCE, *f*-CNF@GCE, and Chitin/*f*-CNF@GCE in 150 μM of 4-MAP recorded in 0.1 M PB (pH 7.0) electrolyte at a sweep rate of 100 mV/s, **[B]** Bar graph of diverse amounts of Chitin/*f*-CNF nanocomposite 2.0 μL–8.0 μL, **[C]** I_p & E_p vs pH for Chitin/*f*-CNF@GCE, **[D]** redox peak current vs. sweep rate **[E]** linear relationship between the anodic peak potential (E□$_a$) and the logarithm of the anodic peak current (log I□$_a$) for the Chitin/*f*-CNF@GCE, and **[F]** E_p Vs. log sweep rate for Chitin/*f*-CNF@GCE.

Table S1. Calculated energy values and reactivity parameters of metol

Parameter	Symbol	Value (eV)	Interpretation
**HOMO energy**	E_{HOMO}	–6.106	Indicates strong electron-donating ability; oxidation is feasible.
**LUMO energy**	E_{LUMO}	–0.851	Suggests potential for accepting electrons during reduction or in redox cycling.
**Band gap**	ΔE	5.255	A moderate gap indicating good stability and moderate reactivity—favorable for selective sensing with sufficient electron transfer efficiency.
**Ionization potential**	IP	6.106	High enough to resist spontaneous oxidation, but suitable for electrode-driven electron transfer.
**Electron affinity**	EA	0.851	Shows metol's ability to accept electrons is limited but possible under electrochemical conditions.
**Chemical hardness**	η	5.680	Indicates moderate resistance to electron exchange—stable but still reactive, aligning with its $2e^{-}/2H^{+}$ redox activity.
**Chemical potential**	μ	–5.680	Negative value confirms strong electron-donating nature, key for oxidation at the electrode.
**Electronegativity**	χ	5.680	Reflects tendency to attract electrons; supports interactions with negatively charged sites or electron-deficient species.
**Electrophilicity index**	ω	91.64	Very high value suggests metol has strong electrophilic character, making it reactive toward nucleophilic species or during redox transformations.
